# Electrically Tunable Lenses for Imaging and Light Manipulation

**DOI:** 10.3390/mi14020319

**Published:** 2023-01-26

**Authors:** Lijun Chen, Shijie Liang, Zhenshi Chen, Xifa Liang, Qingming Chen

**Affiliations:** 1 School of Microelectronics Science and Technology, Sun Yat-sen University, Zhuhai 519082, China; 2 School of Electronic Information, Huzhou College, Huzhou 313000, China; 3 Guangdong Provincial Key Laboratory of Optoelectronic Information Processing Chips and Systems, Sun Yat-sen University, Guangzhou 511400, China

**Keywords:** optofluidics, microfluidics, liquid lens, electrowetting, dielectrophoresis

## Abstract

Optofluidics seamlessly combines optics and microfluidics together to construct novel devices for microsystems, providing flexible reconfigurability and high compatibility. By taking advantage of mature electronic fabrication techniques and flexible regulation of microfluidics, electrically actuated optofluidics has achieved fantastic optical functions. Generally, the optical function is achieved by electrically modulating the interfaces or movements of microdroplets inside a small chamber. The high refractive index difference (~0.5) at the interfaces between liquid/air or liquid/liquid makes unprecedented optical tunability a reality. They are suitable for optical imaging devices, such as microscope and portable electronic. This paper will review the working principle and recent development of electrical optofluidic devices by electrowetting and dielectrophoresis, including optical lens/microscope, beam steering and in-plane light manipulation. Some methods to improve the lens performance are reviewed. In addition, the applications of electrical microfluidics are also discussed. In order to stimulate the development of electrically controlled liquid lens, two novel designs derived from electrowetting and dielectrophoresis are introduced in this paper.

## 1. Introduction

Optofluidics seamlessly combines optics and microfluidics together to develop novel systems for micro-optics applications [1,2,3,4,5,6,7]. By using liquid as the optical medium, it enables the flexible regulation of optical properties and integration with other disciplines, such as biological and medical. Compared with its solid counterpart, optofluidics has some unique characteristics [8,9,10,11], such as wide tunability, better compatibility, small size and low cost, etc. By now, optofluidic lasers [12], tunable waveguides [13,14], reconfigurable optofluidic lenses [15] and electronic paper display [16] have been demonstrated by optofluidics. Among them, the optical lenses and displays deal with light paths for imaging, where the focal length tunability is a key factor that determining its working performance. As the solid lens has constant refractive index (RI) and fixed optical properties, the optical focusing and imaging characteristics are modulated only by physical movement in a conventional optical system. The mechanical component significantly constrains the development of integrated micro system, while in optofluidics, the optical properties can be easily modulated by either changing the lens shape or tuning the refractive index of the liquid. The most straightforward way to change light propagation is tuning the curvature of the refractive interface, which is impossible in a solid medium, while in microfluidics, the interface between immiscible liquids can be easily tuned by pressure control [17], chamber geometry modification [18], or electrical force [19], etc. In addition, the smooth fluidic interface perfectly meets the requirement of optical roughness without significant scattering. By taking advantages of mature electronic fabrication techniques and high compatability with other electronic devices, the electrical modulation is the most promising method for academic and commercial applications. According to the focusing tuning mechanism, electrically optofluidic lenses can be classified as direct electrical actuation, electro-mechanical and electro-thermal coupling. Electro-mechanical actuation usually requires a high voltage of over several hundred volts and the electro-thermal lens has a long response time of several seconds. In terms of the physical properties, the optical medium includes liquid, liquid crystal and elastomer. The liquid crystal lens is polarization dependent, and the elastomer lens has a drawback of high voltage ~1000 volts, while the direct electrical actuation has a better trade-off between voltage requirement (10~100 V) and response speed (~100 ms). In addition, the polarization independent liquid makes it suitable for most of the optical applications. Electrowetting and dielectrophoresis are two key direct electrical actuation methods in optofluidics, where the droplets are precisely manipulated by voltage. In terms of applications, they can be classified as lens, prism and electronic paper, etc. Some commercial electrowetting lenses have found applications in digital cameras or cell phones. Attaching an adaptive liquid lens to a microscope system would increase the depth of field and allows to electrically refocus over a large range of distances. The ability to refocus quickly and precisely leads to many applications such as scanning, package sorting and security. There are some reviews about the electrical optofluidics. Xu gave a comprehensive review of dielectrophoretic optofluidic devices [20]. Fan reported the mechanism of microfluidic droplet manipulations using electrowetting and dielectrophoresis [21]. Chen discussed the recent development of electrically tunable lenses based on the applications for robotics and AI [22]. However, a review about the mechanism of electrical optofluidic lens and its applications on optical imaging and in-plane light manipulation is still required. This review focuses on the electrical optofluidic lens, including the mechanism of electrical droplet manipulation, reconfigurable optical lens, applications on optical imaging and in-plane light manipulation. Specifically, we only discuss the electrical (electrowetting and dielectrophoresis) actuated liquid lens based on the interfacial refraction, where the fluidic interface is electrically modified. 

This article has five sections. The first gives an introduction of optofluidics. Then, electrowetting lens is presented in Section 2, where the application and performance of the lens are discussed. Section 3 is about the dielectrophoretic lens, including the mechanism of dielectrophoresis and out-of-plane/in-plane lenses. Followed by two new electrical lenses derived from electrowetting and dielectrophoresis in Section 4. The last part is a brief conclusion.

## 2. Electrowetting Liquid Lens

### 2.1. The principle of Electrowetting

**Figure 1:**
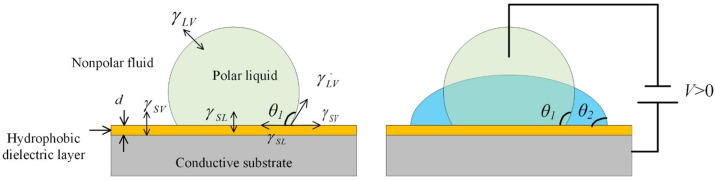
Working principle of electrowetting on dielectrics, (**a**) Small liquid droplet placed on a planar substrate forms a spherical cap due to the influence of surface tension; (**b**) The contact angle is modified by external voltage.

Electrowetting describes the modification of wetting characteristics of a solid surface (usually a hydrophobic one) by applying an external voltage. At first, it is defined as “the change in solid–electrolyte contact angle due to an applied potential difference between the solid and the electrolyte”. To enhance this effect, a dielectric layer is introduced in between the conductive liquid and electrode substrate, which is named electrowetting-on-dielectrics (EWOD), as shown in Figure 1. In addition, gravity effects are neglected in microscale droplets, where the surface tension dominates. In Figure 1a, the initial contact angle $\theta_{1}$ is determined by the interfacial energy between surrounding liquid, polar liquid and the substrate. It is defined by the Young equation [23]:

(1) $$\cos\theta_{1}=\frac{\gamma_{SV}-\gamma_{SL}}{\gamma_{LV}}$$

where $\gamma_{SL}$,$\gamma_{SV}$,$\gamma_{LV}$ are the interfacial surface energies of the solid–polar liquid, solid–liquid medium and polar liquid–liquid medium interfaces, respectively. When an external voltage is applied between the polar liquid and the substrate, the fluidic contact angle will be changed due to the modification of surface tension, see Figure 1b. The relationship between the contact angle and the applied voltage can be described by [23]:

(2) $$\cos\theta_{2}=\frac{\gamma_{SV}-\gamma_{SL}}{\gamma_{LV}}+\frac{\varepsilon }{2\gamma_{LV}d_{}}V^{2}$$

where ε is the dielectric constant of the insulating film, *d* is the thickness of the film, and *V* is the applied voltage. As shown in Figure 1, the shape of the micro droplet can be easily tuned by EWOD. Such a fluidic curvature modification provides the basic mechanism for an optofluidic lens.

### 2.2. Electrowetting Lens and Its Focal Length Tunablity

**Figure 2:**
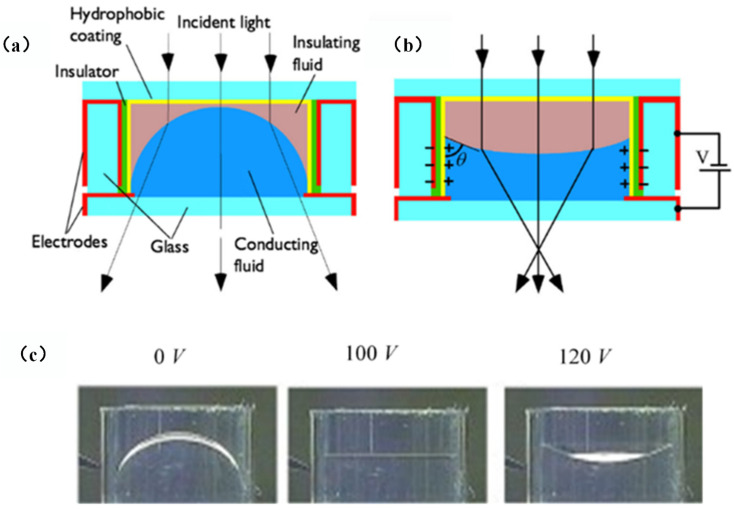
Variable liquid lens actuated by electrowetting [19]. (**a**) Schematic cross section view of the structure. (**b**) When an external voltage is applied between the conductive liquid and the wall, an electric field is exerted on the insulating layer. The contact angle *θ* changes according to the electrowetting effect. (**c**) Experimental results under 0 V, 100 V and 120 V, respectively.

**Figure 3:**
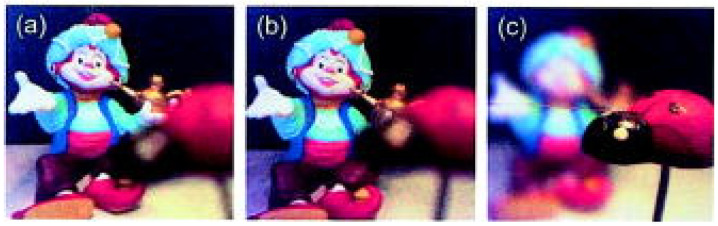
Optical imaging performance [19]. (**a**) Image using a fixed-focus lens. (b) Image with liquid lens focused at 50 cm and (**c**) Image with liquid lens focused at 2 cm.

Electrowetting liquid lens is the most matured electrically adaptive lens, where the focusing properties is reconfigured by tuning surface tension as well as the fluidic curvature of droplet. The simplest one is a plano-convex structure, which focuses the light using a droplet siting on a plane. An early review reported the basic principle and characteristics of electrowetting lens [23]. S. Kuiper et al. proposed a variable-focus liquid lens for miniature cameras using electrowetting [19]. Figure 2 depicts the cross section of the lens. Two immiscible liquids with different refractive indices are filled into a cylindrical container. If the densities of the two liquids are equal, the initial shape of the meniscus is a perfect sphere, which is insensitive to the perturbations and gravitation. To apply an electric field on the side, the cylindrical glass is coated with a transparent electrode (indium tin oxide: ITO), which is then covered by an insulating film. When a voltage is applied between the conductive liquid and the side electrode, there will be an electric field across the insulating layer, see Figure 2b. According to the electrowetting effect, the reduction of the interfacial tension between the conductive fluid and the insulating layer leads to the change of the contact angle between them. Figure 2c shows fluidic interfaces under 0 V, 100 V and 120 V, respectively. The droplet curvature shape changes from convex (convergent lens) to planar, and then to concave (divergent lens). To demonstrate its imaging property, a miniature camera based on the electrowetting lens was developed for imaging. Figure 3b-c show the pictures taken using the liquid lens focused at 50 cm and 2 cm, respectively. For comparison, a similar image was also captured by a fixed-focus lens, see Figure 3a. It is noticed that the camera maintains the good resolution with the liquid lens focusing at 50 cm and 2 cm, and no decrease in performance appeared after over one million switches and experiencing 103 times the Earth’s gravitational constant. This kind of miniature liquid lens camera may find potential applications in mobile electronics and integrated imaging systems.

**Figure 4:**
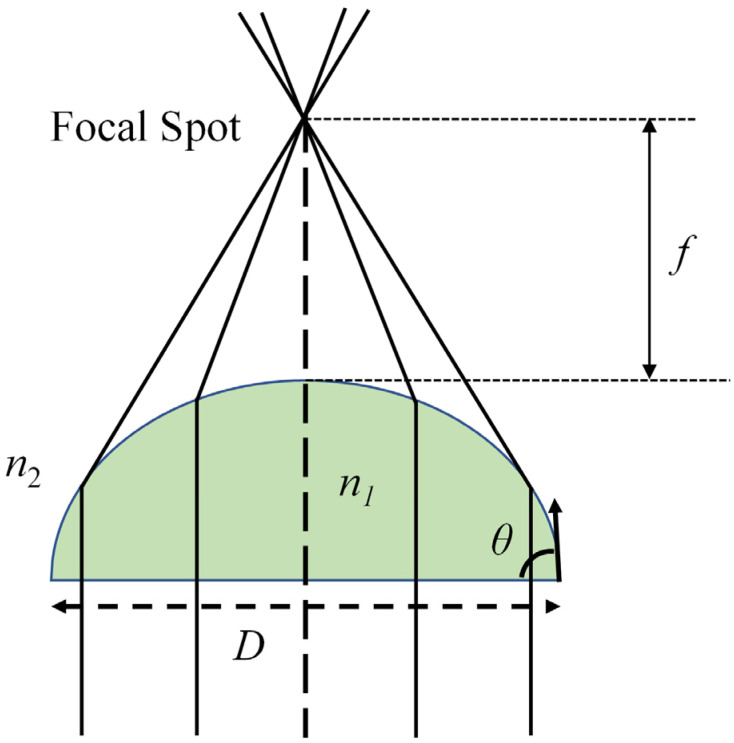
The focusing scheme of a plano-convex liquid lens.

As shown in Figure 3, the tunable focal length of the electrowetting lens makes it able to capture objects located at different positions. The focal length tunability is determined by the interfacial curvature and the refractive index difference at the interface. The focusing scheme of a plano-convex lens structure is shown in Figure 4. The focal length *f* is a function of the diameter of the droplet, contact angle at the substrate and the refractive index difference at the interface. It is described by:

(3) $$f=\frac{D}{2\sin\theta \left(n_{1}-n_{2}\right)}$$

**Figure 5:**
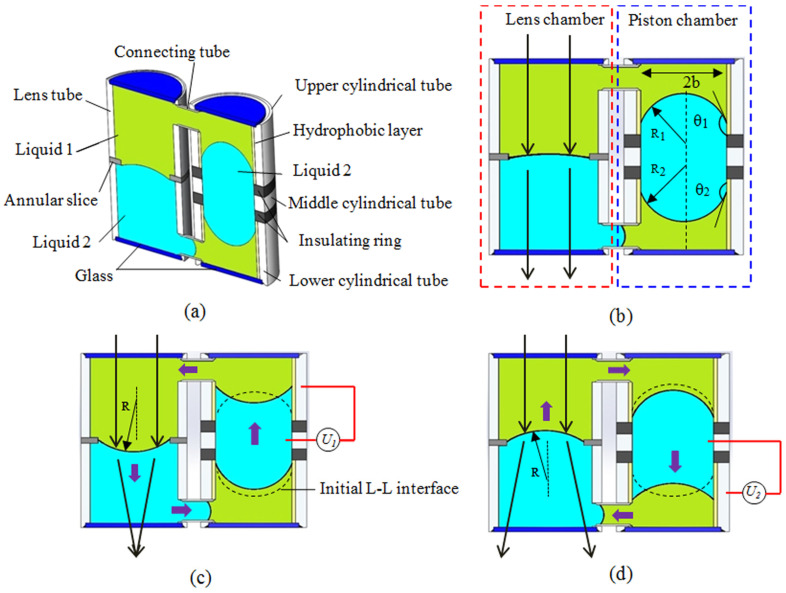
Schematic cross-section view and working principle of the lens with a liquid piston [25]. (**a**) Cell structure and the components. (**b**) Initial equilibrium state. (**c**) The equilibrium state when an extra voltage (*U*_1_) is applied to form a positive lens. (**d**) The negative lens formed by applying an extra voltage (*U*_2_).

In a specific liquid lens, its focal length is tuned by modified the contact angle using the electrowetting effect by external voltage. For example, Krogmann achieved a tunable focal length from 2.3 millimeter to infinite by a voltage of 0~45 V [24]. In this case, the curvature can only be tuned from planar to convex (or positive), which works as a focusing lens. A fluidic interface enables tuning form convex to concave will offer a wide tunable focal length from positive to negative. Li et al proposed an electrowetting lens that continuously varies from positive to negative by using a liquid piston [25], as shown in Figure 5. The structure consists of a piston chamber and a lens chamber, see Figure 5a. In the initial state of Figure 5b, the light goes through the liquid without focusing. Then, a voltage *U*_1_ is applied to the upper electrode of piston chamber, driving the liquid flow along counter-clockwise to form a convex lens for focusing, see Figure 5c. When the external voltage is applied to the lower electrode pair, a concave lens appears in Figure 5d. As the refractive index of the Liquid 1 is greater than that of Liquid 2, a positive lens or a negative lens is formed when a voltage is applied to the upper or lower electrodes. In this case, the shortest negative and positive focal length are around -18 mm and +18 mm, respectively. This is another way to introduce a dielectric film that can sustain a higher voltage, which switches the interface from convex to concave. For instance, the droplet is switched from convex to concave by electrowetting in Figure 2a-b, and Li et al proposed an adaptive lens with the focal length tuning from 15 mm to infinite (~115V), and then to -28 mm (145V) [26]. Although the design shown in Figure 5 provides an effective way to tune the focal length from positive to negative, the requirement of anther liquid chamber increases the size of the lens. In addition, the requirement of one more electrical driver also makes the structure more complex, while the design in Figure 2 has only one liquid chamber and one electrical driver. This simple structure is more suitable for integrated systems, and thus has been widely used in electrowetting lenses.

### 2.3. Imaging Application of Electrowetting Lens

The basic and most important application of lens is imaging, where the refocusing is often used to capture the image of an object. In conventional system, the focusing is usually realized by mechanical movement of lenses, which requires a motor or other types of mechanical drivers. With the development of adaptive lens, it is easy to refocus the imaging system by simply changing the focal length of a liquid lens. This section will give some examples to describe its applications in zooming and microscopy.

**Figure 6:**
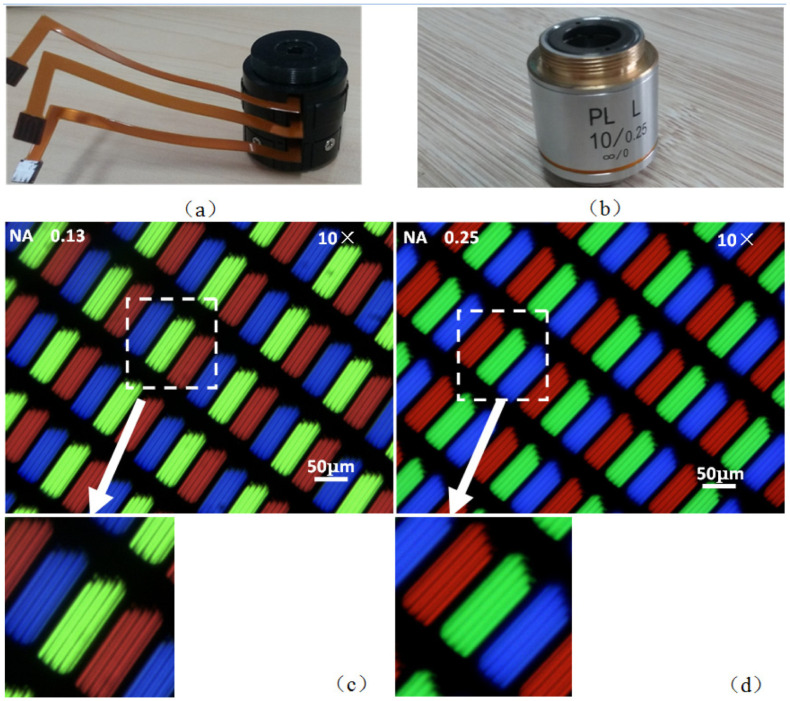
Comparison between the proposed objective and the conventional objective [28]. (**a**) Proposed objective. (**b**) Conventional objective. (**c**) Pixels imaged by proposed objective. (**d**) Pixels imaged by the conventional objective.

An optical zooming system is an optical configuration that can modify the imaging magnification without changing the positions of the object and image, which has been widely used in consumer electronic, telescope, microscopy, and so on [27]. As it requires a tunable focal length and a fixed image plane at the same time, at least two lens groups are used for zooming and focusing. In conventional optical system, the adjustment is achieved by the mechanical movement of solid lenses, making it difficult for a miniature system. The development of the adaptive liquid lens brings the possibility to realize the zooming system without moving parts. The simplest way is to just replace some solid lenses by an adaptive lens, taking the advantages of both solid and liquid lenses. Li et al proposed a zoom microscope objective that could achieve electrically continuous zoom change and correct the aberration at the same time [28]. It consists of three electrowetting liquid lenses and two glass lenses. The liquid lenses provide tunable focal length by an electrowetting effect. In this case, three liquid lenses have the ability to tune the focal power as well as the aberrations. The two glass lenses are used to further increase the focal power and decrease the aberrations, especially the chromatic aberration. The experimental comparison of the optical performance between the proposed objective and a commercial objective (×10) is shown in Figure 6. The observed pixels are captured by the same microscope using these two objectives, respectively. From the results, it is noticed that the imaging quality of the proposed objective is as good as the that of the commercial one, while the proposed objective gets a clearer pixel array (see Figure 6c) because of its dynamic optimizing. It demonstrates the continuous zooming range (~7.8× to ~13.2×) and aberration correction at any wavelength. This work showed the potential imaging application of adaptive lens. 

**Figure 7:**
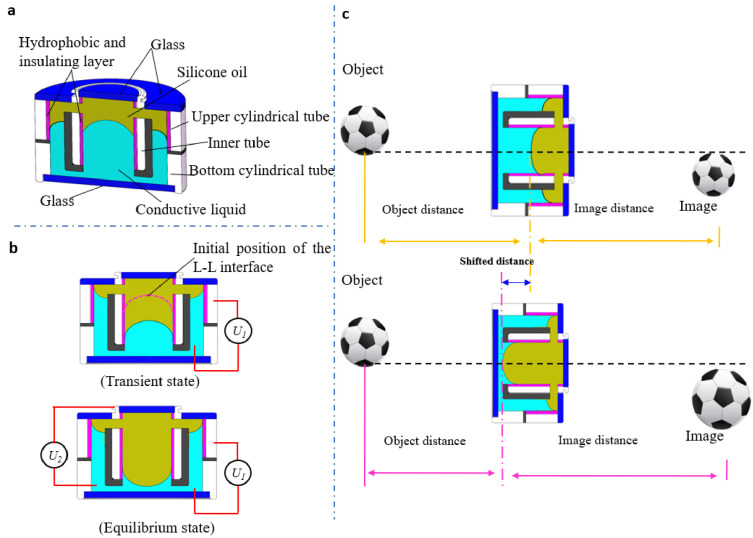
Schematic cross-sectional view and working principle of the displaceable and focus-tunable electrowetting optofluidic lens [29]; (**a**) Cell structure description. (**b**) Moving actuation and deforming actuation of the lens working state. (**c**) Working principle of optical zooming.

However, a single conventional electrowetting lens could not be used as a zoom system because its back focal distance changes while tuning the focal length. Therefore, the lens structure should be changed. To realize an optical zooming system by only one adaptive lens, Li et al proposed an electrowetting lens with a movable fluidic interface [29], as shown in Figure 7. The lens has an annular chamber and a central chamber, with the conductive liquid filled in the bottom, as depicted by Figure 7a. Two electrodes are used to control the position and curvature of the lens, respectively. The actuation process is depicted by Figure 7b. When a voltage *U*_1_ is used to modify the contact angle at the annular chamber, it induces a capillary pressure to push the silicon oil down, thereby displacing the liquid–liquid interface. Then, another voltage *U*_2_ is applied to tune the lens shape as well as its focal length. As the position and focal length of the lens can be tuned independently, it can function as a zoom lens. In Figure 7c, the desired zooming function is demonstrated by well tuning the object and image distance using *U*_1_ and *U*_2_, respectively. The experimental displaceable distance of around 8.3 mm and a zooming ratio of 1.3× are demonstrated in this work [29]. Although a zooming system of single lens has been achieved by tuning the lens position and curvature at the same time, the zoom ratio is only 1.3× due to the limited optical power of one lens, and the single lens is unable to obtain an aberration-corrected image, while by combining the adaptive liquid lens with a solid lens, large zooming ranges (~7.8× to ~13.2×) and great images have been achieved [28]. Its dynamic tunability also increase the fabrication tolerance and decrease the fabrication cost, making it more suitable for practical application.

Some other designs of zooming system by the mix of solid–liquid lenses and liquid lens only, and application cases can be found in Cheng’s review article [27].

**Figure 8:**
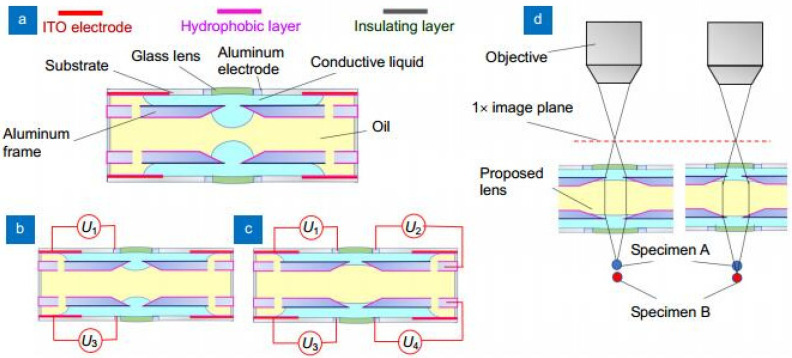
Schematic cross-sectional structure and operating mechanism of the movable electrowetting optofluidic lens [30]. (**a**) Cell structure: the curvature of silicone oil (yellow) and conductive liquid (blue) interface in the central aperture is regulated by external voltage. (**b**) Moving actuation: when the voltages *U*_1_ and *U*_3_ are applied, the liquid interface moves downwards (**c**) Deforming actuation: When external voltage *U*_2_ and *U*_4_ are applied, the liquid interface deforms. (**d**) Working principle of axial scanning.

Optical axial scanning is an essential process to obtain a 3D profile of biological specimens, which requires the refocusing of the microscope. Li et al proposed a movable electrowetting lens for axial scanning microscopy without mechanical movement [30]. The schematic design is shown in Figure 8, which consists of three liquid layers and four external voltages. The yellow silicon oil layer is sandwiched by two conductive liquid layers to form two liquid–liquid (L–L) interfaces, see Figure 8a. As shown in Figure 8b, when two external voltages *U*_1_ and *U*_3_ are applied to the device, the conductive liquid layers pull the two L–L interfaces outwards because of electrowetting effect, and the focal length of the liquid lenses is tuned by applying voltages *U*_2_ and *U*_4_, see Figure 8c. Furthermore, the working principle is displayed in Figure 8d, where the proposed electrowetting lens is used to refocus to specimens A and B located at variable distance along the axial direction. It demonstrates a scanning distance over 1 mm with uniform magnification and great imaging quality.

**Figure 9:**
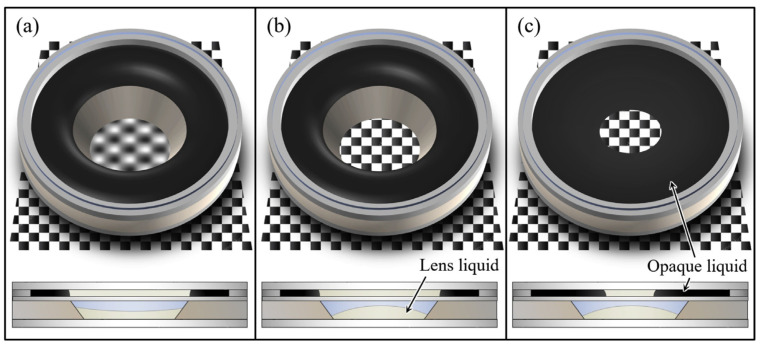
Schematic diagram of multifunctional liquid lens based on EWOD [31]. (**a**) Initial state; (**b**) Variable focusing; (**c**) Variable aperture.

Another EWOD liquid lens set with the tunability of focal length and aperture was first reported by Lee [31]. The schematic design is shown in Figure 9, which consists of a lens unit and an iris unit. When a voltage is applied to modify the curvature as well as the focal length of the lens, the blurred image becomes clear, see Figure 9a-b. Then another voltage drives the opaque liquid shift inward radially, reducing the aperture diameter of the lens set, as shown in Figure 9c. It is suitable for high performance miniature cameras in mobile devices. 

Some other optical functions, such as prisms and lens arrays, have also been demonstrated by electrowetting. For instance, an optical switch based on an electrowetting prism was demonstrated [32]. It provides a large extinction ratio of 47 dB with speeds up to 300 Hz. Another focus-tunable integral imaging system based on an electrowetting lens array is demonstrated by Kim et al [33].

### 2.4. Methods to Enhance the Electrowetting Lens’ Performance

There are some key factors determined the performance of the electrowetting lens, which will be discussed in this section. As for the adaptive lens, the driving mechanism is the first issue need to be settled. In the case of EWOD, the driving voltage, power consumption and response speed should be taken into consideration. 

The EWOD structure is similar to a capacitance, where the energy consumption is resulted from leakage currents, reducing the power dissipation to a very low level. A low power consumption liquid lens was demonstrated by Watson et al [34]. In this work, high-quality parylene AF-4 dielectric layers and large dodecyl sulfate ions are used to reduce the driving voltage to 15V and a recorded low power consumption to tens of microwatts. This will greatly reduce the requirement of power drive in miniature system. There is also an alternative liquid lens system, based on the interface between two immiscible electrolytic solutions (ITIES), which is similar with EWOD. In such a structure, ultra-low driven voltage of ~1V was achieved [35]. Apart from the variable focal length, some other optical properties were also studied to improve the performance of electrowetting lens. To study the astigmatism, Kopp proposed a tubular astigmatism-tunable fluidic lens, which demonstrated back focal length can be tuned by 5 mm and 0° and 45° astigmatism by 3 μm through application of voltages in the range of 50 Vrms [36]. An electrowetting lens with large aperture and focal length tunability was proposed by Song et al [37]. In this study, a triple liquid lens is used to analyze the relations among the focal length, optical aberration and aperture, achieving a root-mean-square wavefront aberration of less than 1/4 waves. Response speed is another key parameter of an adaptive lens. The response time depends on the lens aperture, surface tension and liquid properties (such as density and viscosity). The value is usually within the range of 10 to 100 ms. Supekar proposed an effective way to enhanced response speed of electrowetting lenses with shaped input voltage functions [38]. By combining two exponential driving voltages, they achieved a 29% enhancement when compared to the fastest response obtained using single-exponential driving voltage. Later, in Zhao’s study, it is found that the response time is strongly dependent upon the interfacial surface tension and less dominated by the viscosities [39]. In this study, a shaping driving voltage is used to realize a response time of 22 ms, which is one order-of-magnitude faster response in overdamped lenses. 

## 3. Dielectrophoresis-Actuated Lenses and Applications

### 3.1. Working Principle of Dielectrophoresis

In a neutral particle, the positive core is surrounded by a negative electron cloud. If the particle is placed in a uniform electric field, the nucleus and the electrons will be separated and pushed away in two opposite directions. As a result, the particle is polarized and a dipole moment is induced. The electrostatic force of the positive charge and negative charge is equal but with opposite direction, resulting in force balance inside the particle. The induced dipole moment is *P*=*Qd*, where *d* is the distance between *Q* and -*Q*. The polarized particle will be realigned parallel to the external electric field. If the electric field is nonuniform, the balance state will be broken. When a particle is placed in an inhomogeneous electric field, charge +Q and -Q experience a different electric field. Then, the net force of the dielectric particle can be expressed as [20]:

(4) $${\vec{f}}_{e}=Q\left[\vec{E}\left(r+d\right)-\vec{E}\left(r\right)\right]$$

It is noticed that the force is linear with the gradient of the electric field. It demonstrates that a nonuniform electric field exerts a net force on the dielectric particle. For a bulky object, the polarization force density is used to describe the force intensity. For example, in a homogeneous particle, the dipole density is *Np*. As a result, the dipole moment density is $P=N_{p}\cdot p$. Thus, the electric force density can be written as:

(5) $$\vec{F}=\vec{P}\cdot \nabla \vec{E}$$

where $\vec{F}$ is called Kelvin polarization force density and $\nabla \vec{E}$ is the gradient of electric field. For a liner dielectric material, the polarization density is [20]:

(6) $$\vec{P}=\varepsilon_{0}\chi_{e}\vec{E}=\varepsilon_{0}\left(\varepsilon_{r}-1\right)\vec{E}\;$$

where $\chi_{e}$ is the susceptibility of the material; $\varepsilon_{0}$ and $\varepsilon_{r}$ are the permittivity of free space and the dielectric material, respectively. When the dielectric material is placed in a medium with a dielectric constant of $\varepsilon_{m}$, the Kelvin polarization force density can be rewritten as [20]:

(7) $$\vec{F}=\frac{1}{2}\varepsilon_{0}\left(\varepsilon_{d}-\varepsilon_{m}\right)\nabla \left(\vec{E}\cdot \vec{E}\right)$$

Then, the force exerted on a bulk dielectric object with volume *V* can be calculated using integration over the whole volume. The above equations describe the Kelvin force of a dielectric particle surrounding by a medium with a dielectric constant of $\varepsilon_{m}$. It is noticed that the force depends on the dielectric constant difference as well as the electric field gradient. In addition, the direction of the force is determined by whether the permittivity of the dielectric material is higher or smaller than that of the surrounding medium. 

**Figure 10:**
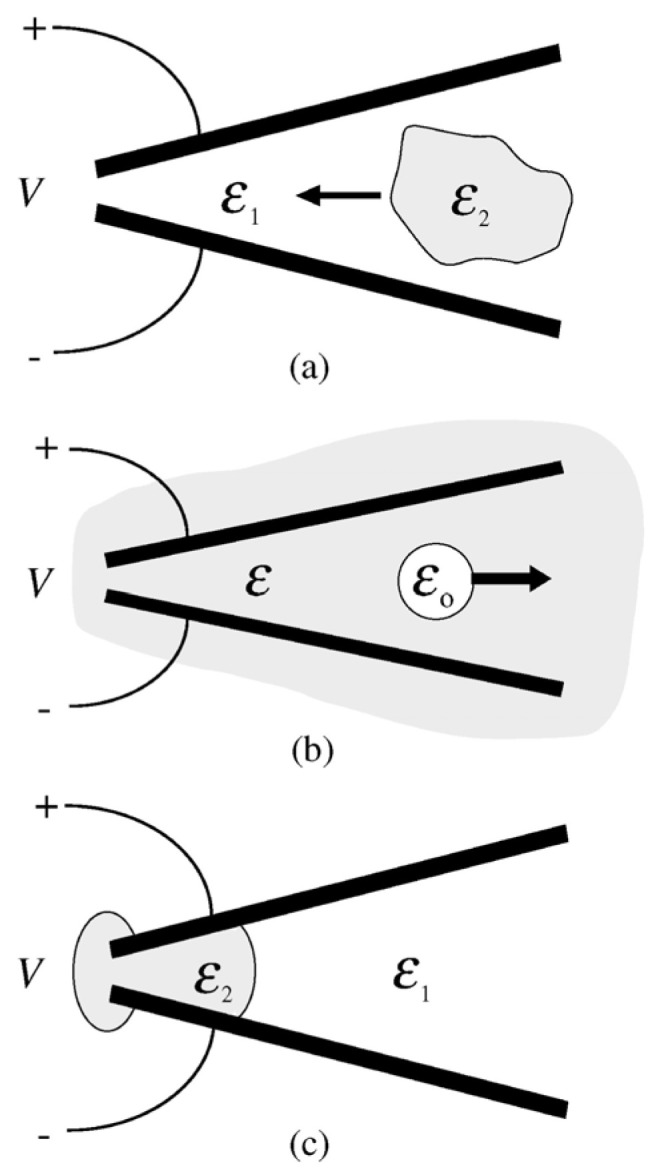
Liquid dielectrophoresis phenomenology [40]: (**a**) Dielectric liquid drawn into strong electric field, *ɛ*_2_ ˃ *ɛ*_1_. (**b**) Bubble repelled from strong electric field, *ɛ* ˃ *ɛ*_0_. (**c**) Control liquid profile with surface parallel to applied electric field, *ɛ*_2_ ˃ *ɛ*_1_.

The phenomenon of the liquid DEP is: dielectric liquid (with higher permittivity) in a non-uniform electric field tends to collect in regions of high electric field intensity [40]. As shown in Figure 10a, DEP force pulls the droplet to the strong electric field. It can also repel the bubble within the liquid from a strong electric field due to the lower permittivity of the bubble; see Figure 10b. In a strong electric field, the free surfaces of the liquid are approximately parallel to the electric field, as shown in Figure 10c. Unlike the electrowetting, the DEP exerts a net force on the fluidic interface.

Fluidic DEP force has been widely used in microfluidic manipulation [41]. Through the electric field induced dielectrophoretic force, the fluidic interface can be dynamically manipulated, resulting in reconfigurable optofluidic devices [20]. Recently, a variety of dielectrophoresis-actuated optofluidic devices, such as liquid lens [42], optical waveguide [43], beam steering [44] and grating [45] have been demonstrated. In terms of the relation between the light and device substrate, optofluidic devices can be divided into out-of-plane and in-plane devices. The former deals with light propagating along the direction perpendicular to the device substrate, which is similar to conventional lens. It can be used to provide tunable focal length in an optical imaging system, while the in-plane device manipulates light propagating along the substrate, which is used for beam manipulation in the microfluidic network.

### 3.2. Dielectrophoresis-Actuated Out-Of-Plane Lens

In the case of an out-of-plane lens, DEP force is used to deform the geometry of a droplet, thereby tuning its focal length. It has some similar properties with an electrowetting lens. For example, they both contain two immiscible liquids and are actuated by external voltages, but the two liquids of DEP lens are insulating, and the electrode structures are more flexible. 

**Figure 11:**
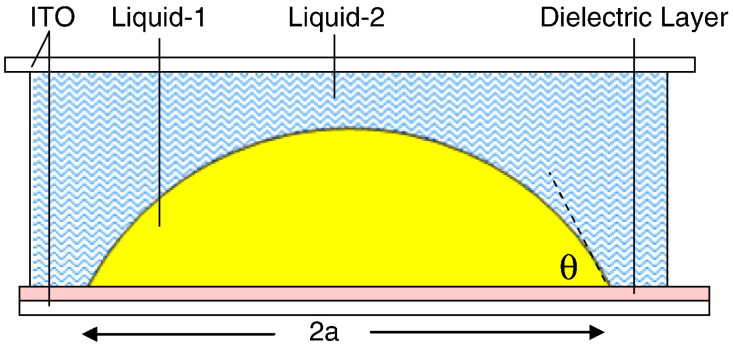
DEP lens with top and bottom electrode [46]. Side view of the lens, the lens is formed by the yellow liquid and surrounded by a blue liquid.

A typical DEP lens with two planar electrodes is shown in Figure 11 [46]. The top and bottom parallel electrodes are used to apply a voltage to generate an electric field across the interface between liquid-1 and liquid-2. As the two liquids with different dielectric constants, a nonuniform electric filed is generated across the droplet. Thus, the droplet is reshaped by the voltage to induce tunable focal length. This is the simplest structure of DEP liquid lens.

**Figure 12:**
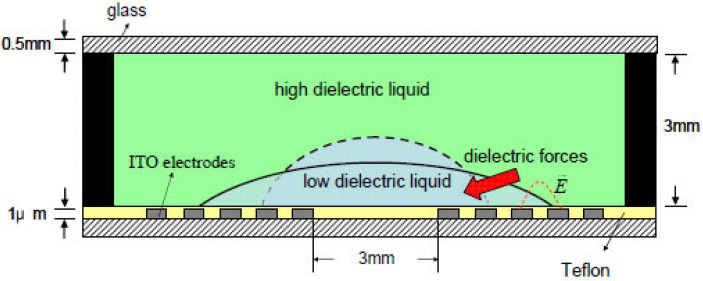
Schematic of dielectric-actuated liquid lens [47]. Under the DEP force, the liquid with higher permittivity shrinks and forces the droplet to a new curvature indicated by the dashed line.

The electrodes of DEP lens can also be placed in the same substrate. Cheng et al demonstrated a dielectrically actuated liquid lens using the DEP [47]. Two immiscible liquids with equal density and different dielectric constants are filled into a PMMA (polymethyl methacrylate) container (Figure 12). Specific concentric electrodes are coated on the bottom glass to apply a nonuniform electric filed on the liquids. As the voltage is turned on, the DEP force drives the liquid with higher permittivity to the strong electric field, thereby deforming the liquid interface and its focal length. While the applied voltage varies from 0 V to 200 V, the focal length is changed from 34 mm to 12 mm. It also demonstrates the focal length tunability by capturing an object placed 50 mm away. Similar DEP-driven liquid lenses have also been demonstrated using liquid crystal [48]. More examples of the DEP-actuated optofluidic devices can be found in Xu’s review [20].

**Figure 13:**
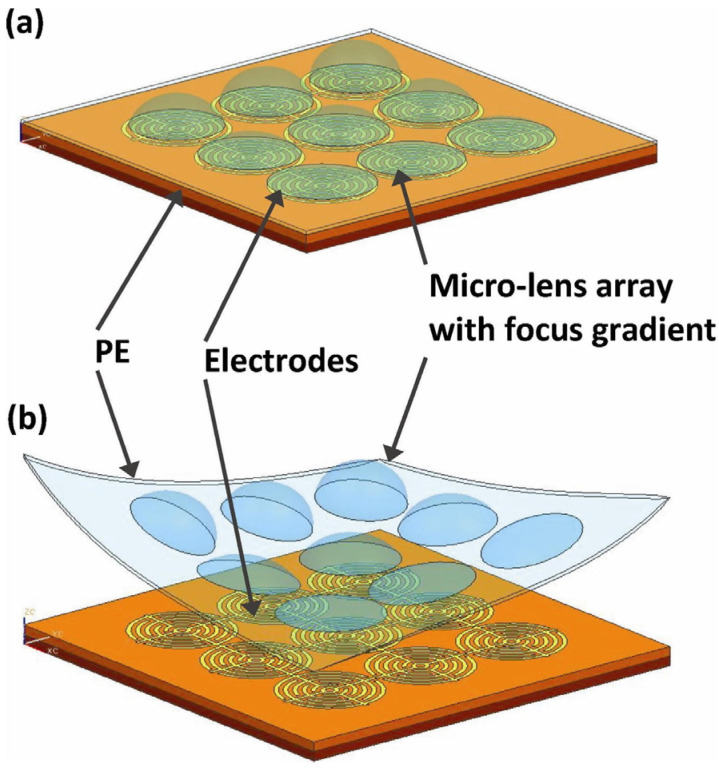
Schematic of the PDMS micro-lens array fabricated by dielectrophoresis [49]. (**a**) Applying variable voltages to concentric circular electrodes for lens array manipulation. (**b**) Peeling the PDMS lens array from substrate.

Benefits from the flexible design of DEP electrode, it enables some adaptive functions that have never been achieved in electrowetting. Wang proposed a method for flexible lens array fabrication using DEP force [49]. As shown in Figure 13a, the concentric circular electrodes are fabricated on the planar substrate for the PEMS droplets manipulation. When variable voltages are applied to the electrodes, it generates a nonuniform electric field for the droplets’ shape modification. Then the curing PDMS lens array is peeled off from the substrate, see Figure 13b. It provides a flexible method to fabricate a lens array with different focal lengths.

The out-of-plane DEP lens has a similar structure to that of electrowetting lens, where the beam is perpendicular to the lens droplet placed on the substrate. As the light passes through the electrodes, only a transparent electrode can be used. The planar electrode structure also provides an easy way to fabricate adaptive lens array. However, its focal length range is limited by the plano-convex lens structure. For instance, the contact angle (*θ*) of the droplet in Figure 11 is unable to vary from positive to negative. When the lens liquid has a higher refractive index than that of the surrounding liquid, only focusing lenses can be realized, while the electrowetting lens of Figure 2 demonstrates the tunable range of focal length from negative to infinite and then to positive.

### 3.3. Dielectrophoresis Actuated Devices for in-Plane Applications

Different from the electrowetting effect, DEP exerts a net force on the fluidic interface to change droplet’s geometry, which enables the application of different types of electrodes in the DEP devices. This section will discuss the DEP actuated in-plane optofluidic device, which is easier for integration of lab-on-a-chip applications. 

A reconfigurable liquid core/liquid cladding waveguide was demonstrated using DEP [43]. Two liquids with different refractive indices are sandwiched by two parallel plates, on which ITO electrodes are coated. By applying a voltage across the plates, the liquid core (with higher RI and higher permittivity) is pumped into the liquid cladding, forming an optical waveguide (*n*_core_ = 1.4341, *ɛ*_core_ = 39; *n*_cladding_ = 1.401, *ε*_cladding_ = 2.5). Both static and moving optical waveguides have been demonstrated in this platform using the DEP driven virtual microchannel. In this case, core liquid is attracted toward the region of strong electric field by the DEP force. This dynamic modulated in-plane optical waveguide is suitable for optical connection and switching in lab-on-a-chip system.

**Figure 14:**
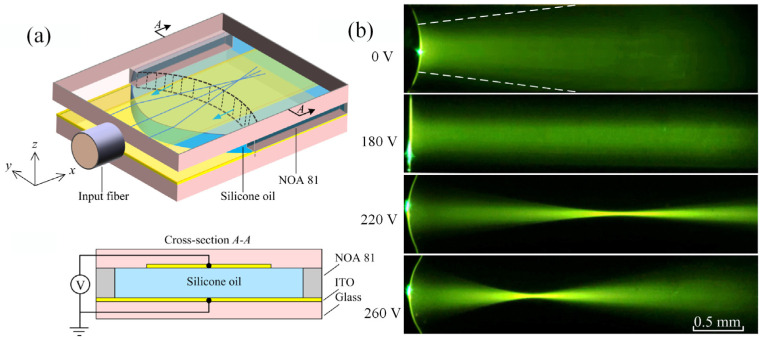
Dielectrophoresis-actuated in-plane liquid lens [51], (**a**) Schematic diagram of top 3D and cross-section view; (**b**) Focusing states under driving voltages of 0 V, 180 V, 220 V and 260 V.

There is another type of liquid lens named in-plane lens, which deals with the light travels along the chip [50]. It promises easier integration with other on chip elements. We reported the first in-plane optofluidic lens by continuously tuning a silicone oil-air interface from concave to convex using the DEP effect [51]. As shown in Figure 14a, it consists of a pair of planar electrodes on top and bottom substrates. When the voltage is applied to the top and bottom electrodes, a DEP force is generated across the liquid–air interface. The in-plane focal length is electrically tuned from about −1 mm to infinite and then from infinite to around +1 mm with the driving voltage of 0 ~ 260 V. The working states under 0 V, 180 V, 220 V and 260 V have been displayed in Figure 14b. It can be seen that well focusing has been achieved. In comparison with electrowetting lens, the in-plane DEP lens is easier to get a wide tunable range covering both negative and positive.

**Figure 15:**
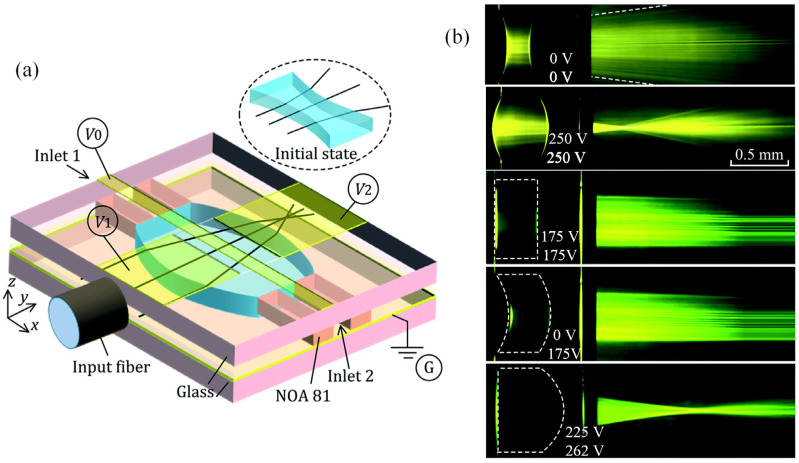
DEP actuated liquid lens with two air-liquid interfaces [52]. (**a**) Schematic design of the dual liquid–air interfaces lens. (**b**) Symmetric and asymmetric focusing states of varied driving voltages (0 V-0 V, 250 V-250 V, 175 V-175 V, 0 V-175 V, 225 V-262 V).

By using an isolated droplet to replace the liquid stream inside a rectangle microfluidic channel, a liquid lens with two air–liquid interfaces is developed [52], as shown in Figure 15a. This two-interface structure further increases the focal length tunable range to 0.5 ~ infinite. As the two top electrodes can be controlled individually, several lensing states, such as double concave (0 V-0 V), double convex (250 V-250 V), planar (175 V-175 V), concave–convex (0V-175 V) and plano–convex (225 V-262 V), have been demonstrated in this work, see Figure 15b. The continuous switching between theses working states in a single droplet greatly enhances the optical performance of lens.

**Figure 16:**
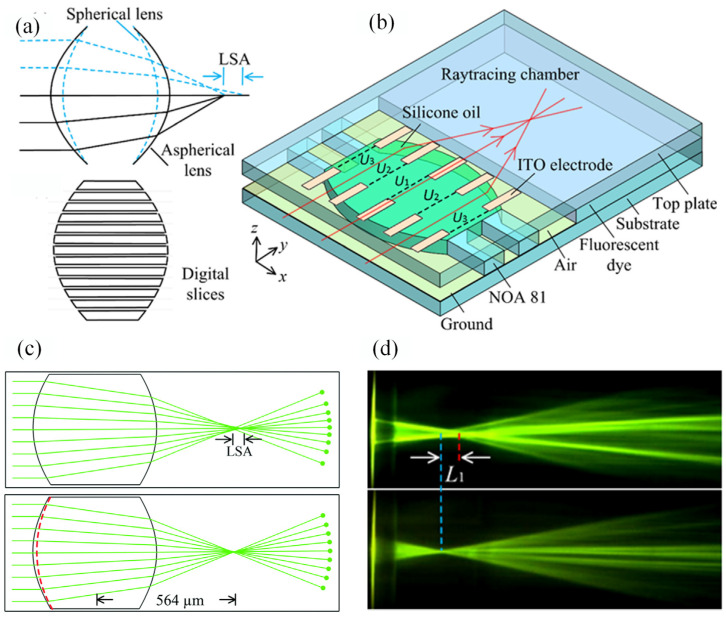
Aberration-free aspherical in-plane tunable liquid lenses by DEP force [53]. (**a**) Schematic design. (**b**) Device design. (**c**) Simulated comparison between spherical and aspherical lenses. (**d**) Experimental comparison between the spherical and aspherical lenses.

Spherical aberration is an important parameter in determining the imaging performance of an optical system. In a solid lens, the aberration is a long-standing problem of fixed focal lenses and a complicated lens set is usually required to compensate for the aberration. In the miniature microfluidic chip, the aberration compensation by a mobile element is not acceptable. The previous spherical optofluidic lenses based on the regulation of global curvature are unable to remedy the aberration. To solve this problem, an in-plane lens with the ability to control the local curvature is proposed [53]. As shown in Figure 16a, the lens geometry is divided into digital slides for local curvature regulation. The movement of each slide is actuated by the DEP force using two planar electrode arrays, see Figure 16b. The two electrode arrays are used to modify the geometry and the focal length of the liquid lens. The digital strip enables the local curvature regulation for aberration compensation. Its aberration is only 1/24 of that in conventional spherical lens, see the simulated and experimental results in Figure 16c and d. It provides a new method for aberration free liquid lens design by using local curvature manipulation. 

These in-plane devices deal with light propagating along the microfluidic chamber, making them more suitable for lab-on-a-chip systems. The reconfigurable optical waveguide can be used for wave guiding in the chip, and the in-plane lens enables the wide range tuning from positive to negative, as well as the switching between different lensing states. In addition, the digital electrode design demonstrates a novel method for the aberration regulation within a single lens.

## 4. Novel Designs for Electrical Liquid Lens

Here, two other types of electrically lenses derived from conventional electrowetting and dielectrophoretic lenses are introduced. The propose of this part is to expand the scope of electrical liquid lenses and provide ideas for new designs of electrical liquid lenses.

**Figure 17:**
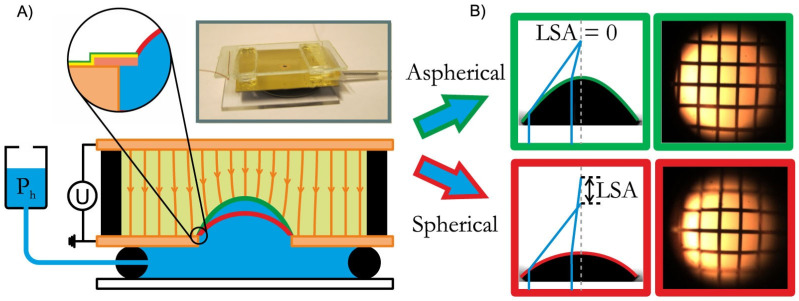
Schematic of tunable lens actuated by hydrostatic pressures and electric fields [54]. (**A**) The curvature of oil (yellow)–water (blue) interface in the central aperture is regulated by a hydrostatic head and a voltage *U* applied between the aperture plate and top electrode. Inset: detail of aperture design for contact line pinning. Top inset: photograph of the actual device. (**B**) Interface profiles of a perfect aspherical lens with zero LSA (top) and of a spherical lens at zero voltage (bottom) along with optical images of a square grid demonstrating the suppression of aberrations.

Conventional adaptive liquid lens suffers from substantial spherical aberration that compromises its imaging quality. To solve this problem, Mishra et al introduced a novel concept of liquid micro-lenses with superior optical performance that allows for simultaneous and independent tuning of both focal length and aberration [54]. As shown in Figure 17A, it consists of three parallel glass plates held by spacers. The space between the two lower plates is filled with water, the one between the upper plates is oil-filled. The oil–water interface is pinned to the edge of the aperture (left inset of a). At zero voltage, the oil–water interface assumes a spherical shape with a curvature determined by hydrostatic pressure, resulting in positive spherical aberration, as shown in Figure 17B. To suppress spherical aberration, the local curvature of the oil–water interface must decrease with increasing distance from the optical axis. When the voltage is applied to the device, it pulls the oil–water interface upward until the electric force is balanced by Laplace pressure. As the distance between the oil–water interface and the top electrode is smaller on the optical axis than elsewhere, E decreases with increasing distance from the optical axis. The curvature of the lens decreases along with it, as required for an aspherical lens. Therefore, an aspherical lens can be achieved by fine tuning the pressure and applied voltage, see Figure 17B. An aspherical lens with variable focal length can be achieved by tuning the pressure and voltage. This work proposes to use hydrostatic pressure to eliminate the aberration of an electrical out-of-plane lens. The perfect aspherical lens demonstrates great imaging quality, which is very important for practical application.

**Figure 18:**
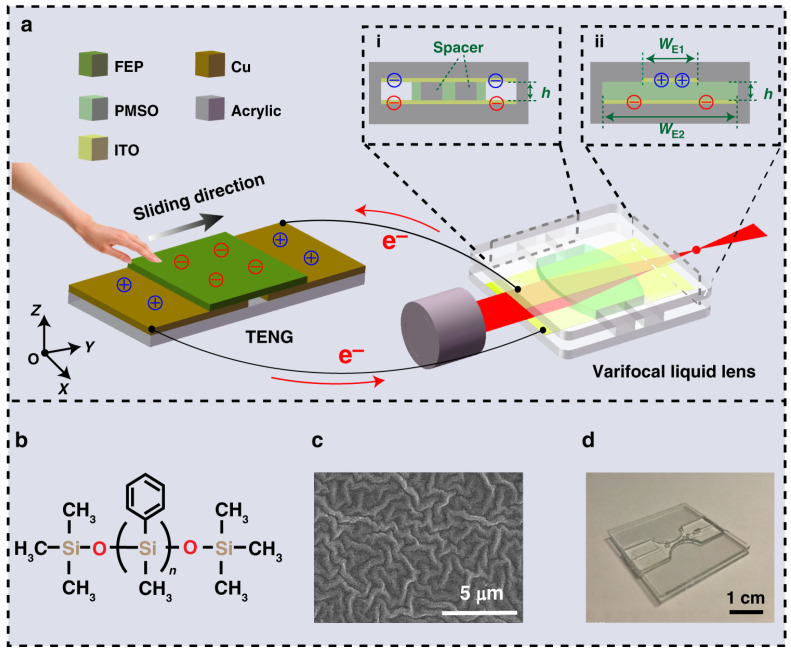
Schematic design of the triboelectric effect-modulated adaptive liquid lens (TVLL) [55]. (**a**) Schematic diagram of the TVLL. (**b**) Molecular structure of the PMSO applied in the TVLL. (**c**) SEM image of the FEP surface with etched micro/nanostructures. (**d**) Photograph of the fabricated TVLL.

In the previous liquid lens, the focal length is tuned by applying external voltage, where an electrical driver is required. Recently, a triboelectric nanogenerator (TENG)-based adaptive lens without the requirement of external voltage is proposed by Fang et al [55]. As shown in Figure 18, it consists of a TENG and a varifocal liquid lens. The TENG is able to convert the tiny mechanical energy into electricity with high voltage and low current, which can be used to drive the capacitive DEP lens. In this work, the focal length is modulated by external mechanical sliding, which generates a dielectrophoretic force by the TENG through the transfer of triboelectric charges in the electrodes. When the mechanical stimulus moves the sliding triboelectric layer, charges accumulating in the electrodes would generate a DEP force to drive the fluidic curvature from biconcave to planar and then to biconvex, tuning the focal length. Figure 18b displays the molecule structure of the optical medium polyphenylmethylsiloxane. To increase the surface area and enhance the TENG effect, the surface of the device is etched by inductively coupled plasma, see Figure 18c. A photograph of the experimental device is shown in Figure 18d. To demonstrate the lensing function, the relationship between the focal length and the sliding distance of TENG was investigated. While the sliding distance increases from 0 to 90 mm, the focal length is tuned from −6 mm to infinite. With a further increase of the distance to 180 mm, the beam is focused to 7 mm. It demonstrates a wide range of focal length adjustment without requiring external drive power. This novel liquid lens may find some applications in lab-on-a-chip systems.

## 5. Conclusions

Adaptive liquid lenses enable wide range tunability of focal length without mechanical movement, and have attracted intensive attention. Among them, the electrical liquid lenses have many merits, such as easy integration, low cost and no requirement of continuous liquid supply or external pumping. In addition, some the electrical lenses have been commercialized. In this paper, we have reviewed the two most widely used electrical lenses: electrowetting lens and dielectrophoretic lens. The driving mechanisms and working principles of these two types of lenses have been discussed. The electrowetting lens is suitable to replace solid lenses in conventional imaging systems, providing tunable focal length and zooming ratio. Some classical cases of zoom system and microscopy imaging using electrowetting lenses are discussed. In terms of dielectrophoretic lens, the flexible design of electrode makes it suitable for micro-lens array and in-plane light manipulation. Some recently developed in-plane electrical lenses are introduced. Some key parameters, such as power consumption, response time and aberration, have also been discussed. The electrowetting lens has a wide range tunable focal length from positive to negative. However, the out-of-plane dielectrophoretic lens only works in the plano-convex state. Both of them have a low power consumption and the same voltage range of 10~100 volts. However, the electrowetting lens has a response time of 10 to 100 milliseconds, which is about an order faster than that of dielectrophoretic lens. Therefore, the wide focusing range and rapid response make electrowetting lens have more potential in imaging systems. The dielectrophoretic lenses are suitable for lab-on-a-chip application and promise higher scalability. At last, two examples of new adaptive lens design are presented to expand the scope of the electrical lens.

## Data Availability

Not applicable.
